# Buccal versus lingual mucosal grafts for anterior urethral stricture management: A prospective surgical outcome and morbidity comparison

**DOI:** 10.14440/bladder.2024.0063

**Published:** 2025-05-05

**Authors:** Ahmed Moustafa Nafie, Mahmoud Moustafa Nafie, Ahmed Aosmali, Basma Mohamed Soliman

**Affiliations:** 1 Department of Urology, Frimley Park Hospital, Frimley, Surrey GU16 7UJ, England; 2 Department of General Surgery, King’s College Hospital, London SE5 9RS, England; 3 Department of Emergency, King’s College Hospital, London SE5 9RS, England

**Keywords:** Anterior urethral stricture, Donor site morbidity, Buccal mucosal graft, Lingual mucosal graft, Oral complications, Stricture recurrence, Graft success

## Abstract

**Background:**

Urethral stricture is characterized by long-term scarring and narrowing of the urethral canal caused by acute trauma, inflammation, or medical procedures, such as urethral instrumentation or surgery. Despite the widespread use of both buccal and lingual mucosal grafts (LMG) in urethroplasty, few prospective studies have directly compared their surgical outcomes and donor site morbidity. This study aims to fill that gap.

**Objective:**

This study compares the use of buccal and LMG in managing anterior urethral stricture with surgical outcomes and donor site morbidity evaluations.

**Methods:**

This case–control comparative study was conducted at Ain Shams University Hospital. Patients who attended the urology outpatient clinic, presenting with lower urinary tract symptoms secondary to stricture anterior urethra and underwent surgical management by urethroplasty with a dorsal onlay technique, were selected as cases.

**Results:**

No statistically significant differences were observed between the studied groups regarding age, smoking status, comorbidities, related urinary conditions, or the presence of a urinary catheter. In addition, the groups had no significant differences concerning stricture characteristics, graft details, or operation specifics. Similarly, general and urethral outcomes showed no statistically significant variation between the groups. Problems with drinking, soft food consumption, solid food consumption, dysgeusia, and speaking were significantly less frequent in the buccal mucosal graft (BMG) group than in the LMG group. In contrast, oral tightness was significantly more frequent in the BMG group than in the LMG group.

**Conclusion:**

The study concluded that buccal and LMG effectively repair anterior urethral stricture, showing similar success rates. However, LMG patients experience earlier oral complications, while BMG patients face more long-term oral tightness, making graft choice dependent on patient-specific tolerances.

## 1. Introduction

Urethral stricture involves the chronic scarring and narrowing of the urethral passage, often resulting from acute injuries, inflammatory conditions, or medical procedures, such as surgery and instrumentation within the urethra. In males, urethral strictures commonly lead to urinary tract obstructions, which can manifest as lower urinary tract symptoms, recurrent infections, bladder dysfunction, and, occasionally, kidney damage. Treatment goals for urethral strictures focus on relieving obstruction and mitigating lower urinary tract symptoms.^1^

Current research increasingly favors urethroplasty over repeated visual internal urethrotomy for treating anterior urethral strictures. This preference stems from the high recurrence rates associated with endoscopic treatments and the cost-effectiveness of urethroplasty as a more permanent solution.^2^

Graft urethroplasty is a well-established technique for penile and bulbar urethral strictures that cannot be treated with anastomotic repair. Urethral reconstruction using a buccal mucosal graft (BMG) to substitute the urethral mucosa has become a well-established modality in the management of bulbar and penile urethral strictures, not amenable to excision and anastomosis.^3^ Given that the lateral and ventral tongue mucosa share similar histological properties with other mucosal surfaces, the lingual mucosal graft (LMG) was introduced by Simonato *et al*.^4^ in 2006 as an alternative. Early studies on LMGs reported minimal local morbidity, but comparisons with buccal graft outcomes were limited.^4^ This study, therefore, aims to compare buccal and LMG in terms of surgical success and donor site impacts in treating anterior urethral strictures.

## 2. Patients and methods

After ethical committee approval and written consent from the patients, a prospective case–control study was carried out at the Ain Shams University Hospital from February 2020 to January 2022. All cases were selected from those attending the urology outpatient clinic presenting with lower urinary tract symptoms secondary to stricture anterior urethra and prepared for surgical management by urethroplasty with dorsal onlay technique. This study was approved by the Institutional Review Board, Research Ethics Committee, Faculty of Medicine, Ain Shams University (FWA 000017585; Approval Number: MD65/2020).

### 2.1. Inclusion criteria

Patients were selected for the study if they had long anterior urethral strictures requiring substitution urethroplasty (more than or equal to 3 cm).

### 2.2. Exclusion criteria

Patients were excluded if they had a history of oral surgeries, noticeable diseases or changes in the oral mucosa, limitations in mouth opening, and tongue-tie (reduced tongue tip mobility caused by a shortened frenulum). Moreover, patients with neurological lesions causing neuropathic disorders, such as neurogenic bladder and atonic bladder, and patients with short urethral stricture (<2.5 cm) or cases with posterior stricture urethra were not included in the study.

### 2.3. Participant assignment

Patients were randomly assigned to undergo urethroplasty using BMG or LMG, with 30 patients in each group. The study followed a prospective case–control design, ensuring that the allocation of patients to each surgical group was unbiased. However, if a patient had unhealthy buccal mucosa (e.g., due to tobacco use or oral conditions), they were shifted to the LMG group instead of BMG. The division allowed for a comparative analysis of surgical outcomes and donor site morbidity between the two graft types.

### 2.4. Study procedures

All participants underwent the following procedures:

(i) Careful history-taking regarding personal, medical, and surgical histories.
(ii) Complete physical examination to exclude any disorders that may interfere with the results, including hip mobility and perineal area, and careful assessment of oral and lingual mucosa.
(iii) Oral hygiene: Patients were instructed to use 5% povidone-iodine mouth gargles thrice daily, starting 48 h before surgery.
(iv) Pre-operative radiological investigations, including uroflowmetry and American Urological Association (AUA) symptom score assessment and abdominopelvic ultrasound with pre- and post-void residual urine.
(v) Retrograde urethrogram (RGU), voiding cystourethrogram (VCUG), and cystourethroscopy to comprehensively evaluate the urinary tract, including assessing the bladder and kidneys for secondary effects of the urethral stricture – such as hydronephrosis, bladder wall thickness, or residual urine volume after voiding.
(vi) Pre-operative laboratory investigations, including hemoglobin levels, kidney function tests, and urine culture and sensitivity.

### 2.5. Steps of intervention for both groups

The operations were performed by a team comprised of two well-trained lecturers and two associate professors, all with 5 – 10 years of experience in this specific type of surgery. A consistent surgical team conducted all the procedures to maintain uniformity and reduce variability in surgical technique. The surgical procedure performed was a single-stage onlay graft urethroplasty.

#### 2.5.1. Graft length measurement

The length of the urethral stricture was assessed preoperatively using RGU, VCUG, and urethroscopy. The required graft length was determined during surgery by measuring the length of the strictured urethral segment after complete mobilization and urethrotomy. The harvested graft was tailored to match the measured length and width of the opened urethrotomy, ensuring a tension-free placement.

#### 2.5.2. Urethral mobilization

The urethral mobilization was conducted using the perineal approach and dorsal urethrotomy. In the perineal approach, the strictured segment of the urethra was completely mobilized. In distal penile urethra cases, a circum-coronal incision was used. The mobilized urethra was rotated at 180°. A dorsal urethrotomy was performed on the dorsal aspect of the urethra and extended into the healthy urethra, ensuring a 1 cm margin on both ends.

#### 2.5.3. Graft harvesting

For BMG, the graft was harvested from the inner cheek or lower lip (if additional graft length was needed). The edges were infiltrated with 2% lignocaine with adrenaline for hemostasis, and the graft site was closed with 4 – 0 polyglactin sutures. For LMG, the graft was harvested from the lateral aspect of the tongue, and the full-thickness graft was obtained using traction and a mouth opener. The graft was tailored and defatted to remove any submucosal fibrovascular tissue or muscle fibers.

#### 2.5.4. Graft placement

The harvested graft was sutured onto the dorsal aspect of the urethra. It was fixed to the corpora cavernosa in the midline using intermittent 4 – 0 polyglactin sutures. The urethral epithelium was coated with continuous running sutures over a 16–French silicon Foley catheter.

#### 2.5.5. Bilateral graft harvesting

Bilateral graft harvesting was necessary in cases where the length of the stricture exceeded the typical length that could be harvested from a single donor site. Specifically, in single-site limitations for BMG, a single cheek typically provides up to 6 cm of graft. If a longer graft was required, the contralateral cheek or the lower lip was used as an additional source.^5^,^6^ For LMGs), the lateral aspect of one side of the tongue could provide grafts of up to 7 – 8 cm. For longer grafts, harvesting extended across the tip of the tongue to the contralateral side.^5^,^6^ Patients with longer strictures (e.g., pan-urethral strictures or those exceeding 10 cm in length) often needed bilateral harvesting to ensure a sufficient length and width graft for effective reconstruction.

### 2.6. Post-operative care

#### 2.6.1. Immediate post-operative management

The immediate post-operative management consisted of pain management with diclofenac injections and oral tablets and continuation of mouth gargles for oral hygiene. No dietary restrictions were imposed post-surgery.

#### 2.6.2. Catheter management

The urethral catheter was removed 3 weeks after confirmation of no contrast extravasation in pericatheter urethrography. If extravasation was noted, the catheter was retained for another 2 weeks.

#### 2.6.3. Post-operative follow-ups

For surgical outcomes, patients were evaluated at 3 – 6 months post-operation using uroflowmetry, RGU + VCUG, and AUA symptom scores. A pericatheter urethrogram was conducted at 3 weeks post-operation to evaluate the healing of the urethra and at 3 – 6 months post-operation to check patency. Long-term follow-up involved symptom assessment and uroflowmetry every 6 months, with repeat urethrography for suspected recurrence. In addition, uroflowmetry and the AUA symptom score were evaluated 3 months post-surgery, with failure defined as stricture recurrence or fistula necessitating further intervention.^1^

Pain in the oral cavity was evaluated on post-operative day 3 using a numeric rating scale (0 = *No pain* to 10 = *Worst possible pain*), analgesic needs, and a visual analog scale to monitor donor site morbidity. Oral morbidity was also examined via a home questionnaire, assessing issues with drinking and eating (options: *No problem*, *slightly difficult*, *very difficult*, or *impossible*) and addressing speech, sensitivity, and taste disturbances through yes or no questions. Pain and oral function were reassessed at 2 weeks and 6 months post-operation, using the numeric rating scale for pain and yes or no questions to gauge difficulties with drinking, eating (soft and solid foods), oral tightness, sensory changes, salivation, speech, and taste.

#### 2.6.4. Methods of managing the area from which the graft was harvested

In an open donor site, the donor site is left open to heal naturally without sutures after graft harvesting. For a closed donor site, the donor site is sutured closed after graft harvesting, which may minimize bleeding and accelerate initial healing but could lead to increased post-operative tightness or discomfort.

### 2.7. Outcome measures

Outcome measures included an estimation of operative time, intraoperative complications, early post-operative complications, catheter time, and hospital stay.

### 2.8. Sample size

The required sample size was calculated using the Power Analysis and Sample Size Software version 11 (NCSS, LLC., United States), employing a two-sample *t*-test with pooled variance. The significance level was set at *p*<0.05, corresponding to a 95% confidence level.

### 2.9. Ethical considerations

All patient data were anonymized, with presentations based solely on diagnosis rather than names, ensuring the protection of patient confidentiality. Informed consent was obtained from each participant and documented in Arabic with date and time confirmation. Confidentiality was maintained by assigning a unique number to each patient’s initials, which only the investigator could access.

### 2.10. Statistical analysis

Data collected were coded, organized, and statistically evaluated using IBM Statistical Package for Social Sciences software (version 28.0, IBM Corp., United States). Quantitative results were expressed as mean ± standard deviation, along with the range’s minimum and maximum values, and analyzed using an independent *t*-test. Qualitative data were presented as counts and percentages, with comparisons made through the Chi-square and Fisher’s exact tests. Statistical significance was set at *p*≤0.05, with higher values deemed non-significant.

## 3. Results

**Figure 1:**
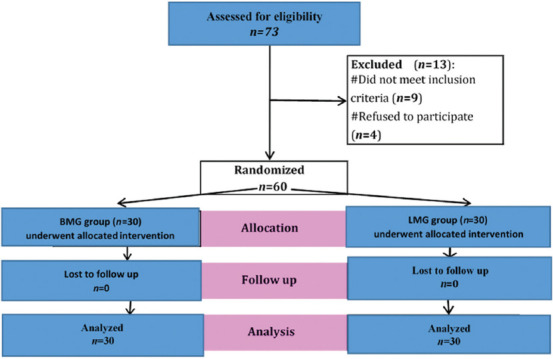
Flow chart of the studied cases Abbreviations: BMG: Buccal mucosal graft; LMG: Lingual mucosal graft.

During this study, 73 patients were assessed for eligibility, and 60 patients were included in the study (30 in each group). Of all eligible patients, nine were excluded from the study based on the inclusion criteria, and four refused to participate (Figure 1). Ultimately, the analysis was performed based on the data of 60 patients who presented with lower urinary tract symptoms due to anterior urethral stricture.

Table 1 presents the demographic and baseline characteristics of the study population. There were no statistically significant differences between the BMG and LMG groups in terms of age, smoking status, comorbidities, associated urinary conditions, or urinary catheter use. The mean age of patients in the BMG group was 38.6 ± 12.4 years, compared to 39.8 ± 11.8 years in the LMG group (*p*=0.694). The prevalence of smoking was slightly higher in the BMG group (36.7%) compared to the LMG group (33.3%), but this difference was not significant (*p*=0.787). In addition, hypertension and diabetes were more common in the BMG group (30% and 13.3%, respectively) than in the LMG group (23.3% and 10%), but these differences were not statistically significant (*p*=0.559 and *p*=0.999, respectively). The presence of urinary infections was higher in the LMG group (10%) than in the BMG group (6.7%), though this difference was also not significant (*p*=0.999).

**Table 1:** Demographic characteristics between the studied groups

Variables	BMG group, *n* (%)	LMG group, *n* (%)	*p*-value
Age (years)	38.6±12.4	39.8±11.8	0.694
Smoking	11 (36.7)	10 (33.3)	0.787
Hypertension	9 (30.0)	7 (23.3)	0.559
Diabetes mellitus	4 (13.3)	3 (10.0)	0.999
Urinary infection	2 (6.7)	3 (10.0)	0.999
Urinary catheter	2 (6.7)	1 (3.3)	0.999

Abbreviations: BMG: Buccal mucosal graft; LMG: Lingual mucosal graft.

Table 2 details the characteristics of the urethral strictures, including their location, length, graft dimensions, and key surgical parameters. There was no significant difference between the two groups regarding stricture length, which was slightly longer in the LMG group (8.3 ± 3.1 cm) than in the BMG group (7.6 ± 3.3 cm) (*p*=0.408). The stricture location varied slightly, with penile strictures being the most common in both groups (56.7% in BMG vs. 60% in LMG), followed by bulbar strictures (30% in BMG vs. 23.3% in LMG) and penobulbar strictures (13.3% in BMG vs. 16.7% in LMG) (*p*=0.876). The etiology of strictures was similar, with trauma being the predominant cause (80% in BMG vs. 76.7% in LMG) and idiopathic causes accounting for the remainder (*p*=0.754).

**Table 2:** Stricture, graft, and operation characteristics between the studied groups

Variables	BMG group, *n* (%)	LMG group, *n* (%)	*p*-value
Stricture length (cm)	7.6±3.3	8.3±3.1	0.408
Stricture location			
Bulbar	9 (30.0)	7 (23.3)	0.876
Penile	17 (56.7)	18 (60.0)	
Penobulbar	4 (13.3)	5 (16.7)	
Stricture etiology			
Trauma	24 (80.0)	23 (76.7)	0.754
Idiopathic	6 (20.0)	7 (23.3)	
Graft length (cm) before surgery	8.1±3.3	8.7±3.1	0.425
Graft width (cm) before surgery	2.1±0.4	2.1±0.4	0.896
Bilateral graft harvesting	5 (16.7)	6 (20.0)	0.739
Donor site			
Open	11 (36.7)	9 (30.0)	0.584
Closed	19 (63.3)	21 (70.0)	

Abbreviations: BMG: Buccal mucosal graft; LMG: Lingual mucosal graft.

Regarding graft dimensions, the mean graft length before surgery was greater in the LMG group (8.7 ± 3.1 cm) compared to the BMG group (8.1 ± 3.3 cm) (*p*=0.425). The graft width was identical in both groups (2.1 ± 0.4 cm, *p*=0.896). Bilateral graft harvesting was required in a few cases, affecting 16.7% of BMG patients and 20% of LMG patients (*p*=0.739). In terms of donor site closure, 36.7% of BMG cases and 30% of LMG cases left the donor site open, while the rest underwent surgical closure (*p*=0.584).

Table 3 compares the surgical outcomes between the two groups. There were no significant differences in operative time, hospital stay, or catheter duration, suggesting that the type of graft did not impact perioperative recovery. The mean operation duration was nearly identical, at 117.2 ± 32.6 min in BMG and 118.7 ± 35.6 min in LMG (*p*=0.868). Similarly, the hospital stay was shorter in the LMG group (4.8 ± 1.1 days) than in the BMG group (5.1 ± 1.2 days), but the difference was not statistically significant (*p*=0.307). The catheter duration was also comparable between groups, with a mean of 14.9 ± 3.8 days in BMG and 15.2 ± 3.8 days in LMG (*p*=0.762).

**Table 3:** General and urethral outcomes between the studied groups

Variables	BMG group, n (%)	LMG group, n (%)	*p*-value	Relative effect	

				Mean±standard deviation/relative risk	95% confidence interval
Operation duration (minutes)	117.2±32.6	118.7±35.6	0.868	-1.5±8.8	-19.1 – 16.2
Hospital stay (days)	5.1±1.2	4.8±1.1	0.307	0.3±0.3	-0.3 – 0.9
Catheter stay (days)	14.9±3.8	15.2±3.8	0.762	-0.3±1.0	-2.3 – 1.7
Post-operative urinary flow (mL/second)	16.4±6.4	18.3±6.5	0.260	-1.9±1.7	-5.2 – 1.4
Failure (recurrence)	4 (13.3%)	2 (6.7%)	0.671	2.00	0.40 – 10.11

Abbreviations: BMG: Buccal mucosal graft; LMG: Lingual mucosal graft.

Post-operative urinary flow (Q_max_) improved significantly in both groups, indicating successful urethral reconstruction. The mean post-operative Q_max_ was 16.4 ± 6.4 mL/s in the BMG group and 18.3 ± 6.5 mL/s in the LMG group, with no statistically significant difference (*p*=0.260). The recurrence rate of urethral stricture was 13.3% in BMG and 6.7% in LMG, though this difference was insignificant (*p*=0.671).

Table 4 tabulates the post-operative donor site morbidity, comparing pain levels, eating and drinking difficulties, speech problems, and other oral complications. Early post-operative complications were more frequent in the LMG group, while long-term complications were more common in the BMG group.

**Table 4:** Oral complications between the studied groups

Complications	BMG group, *n* (%)	LMG group, *n* (%)	*p*-value	Relative effect	

				Relative risk	95% confidence interval
Numeric rating scale >3					
Day-3	20 (66.7)	21 (70.0)	0.781	0.95	0.67 – 1.34
Week-2	12 (40.0)	14 (46.7)	0.602	0.86	0.48 – 1.53
Month-6	2 (6.7)	4 (13.3)	0.671	0.50	0.10 – 2.53
Problems with drinking					
Day-3	3 (10.0)	11 (36.7)	0.015*	0.27	0.08 – 0.88
Week-2	2 (6.7)	6 (20.0)	0.254	0.33	0.07 – 1.52
Month-6	1 (3.3)	3 (10.0)	0.612	0.33	0.04 – 3.03
Problems with eating soft food					
Day-3	6 (20.0)	14 (46.7)	0.028*	0.43	0.19 – 0.96
Week-2	3 (10.0)	5 (16.7)	0.706	0.60	0.16 – 2.29
Month-6	1 (3.3)	4 (13.3)	0.353	0.25	0.03 – 2.11
Problems with eating solid food					
Day-3	11 (36.7)	19 (63.3)	0.039*	0.58	0.34 – 1.00
Week-2	6 (20.0)	14 (46.7)	0.028*	0.43	0.19 – 0.96
Month-6	3 (10.0)	6 (20.0)	0.472	0.50	0.14 – 1.82
Oral tightness					
Day-3	20 (66.7)	8 (26.7)	0.002*	2.50	1.31 – 4.77
Week-2	13 (43.3)	4 (13.3)	0.010*	3.25	1.20 – 8.83
Month-6	8 (26.7)	2 (6.7)	0.038*	4.00	0.92 – 17.30
Sensitivity disorders					
Day-3	23 (76.7)	19 (63.3)	0.260	1.21	0.86 – 1.69
Week-2	17 (56.7)	13 (43.3)	0.302	1.31	0.78 – 2.19
Month-6	11 (36.7)	7 (23.3)	0.260	1.57	0.71 – 3.50
Salivatory disorders					
Day-3	11 (36.7)	15 (50.0)	0.297	0.73	0.41 – 1.32
Week-2	6 (20.0)	9 (30.0)	0.371	0.67	0.27 – 1.64
Month-6	2 (6.7)	4 (13.3)	0.671	0.50	0.10 – 2.53
Dysgeusia					
Day-3	4 (13.3)	19 (63.3)	<0.001*	0.21	0.08 – 0.55
Week-2	3 (10.0)	12 (40.0)	0.007*	0.25	0.08 – 0.80
Month-6	1 (3.3)	5 (16.7)	0.195	0.20	0.02 – 1.61
Speaking problems					
Day-3	10 (33.3)	26 (86.7)	<0.001*	0.38	0.23 – 0.65
Week-2	6 (20.0)	16 (53.3)	0.007*	0.38	0.17 – 0.83
Month-6	2 (6.7)	9 (30.0)	0.020*	0.22	0.05 – 0.94
Oral bleeding					
Day-3	0 (0.0)	0 (0.0)	NA	NA	NA
Week-2	0 (0.0)	0 (0.0)	NA	NA	NA
Month-6	0 (0.0)	0 (0.0)	NA	NA	NA

Note:

* Refers to statistically significant values determined at *p≤*0.05. Abbreviations: BMG: Buccal mucosal graft; LMG: Lingual mucosal graft; NA: Not available.

On post-operative day 3, 86.7% of LMG patients reported speech problems, compared to only 33.3% in the BMG group (*p*<0.001). Similarly, dysgeusia (taste disturbances) was significantly higher in the LMG group (63.3%) than in the BMG group (13.3%) (*p*<0.001). Problems with drinking were 3 times more common in the BMG group (36.7%) compared to the LMG group (10%) (*p*=0.015). Eating difficulties followed a similar trend, with problems eating soft food reported by 46.7% of LMG patients versus 20% among BMG patients (*p*=0.028).

Despite these early complications in LMG patients, long-term donor site complications were more frequent in BMG patients. By 6 months post-operation, 26.7% of BMG patients continued to experience oral tightness, compared to only 6.7% in the LMG group (*p*=0.038). Persistent sensitivity disorders were also more common in the BMG group (36.7%) compared to 23.3% in LMG (*p*=0.260), although this difference was not statistically significant.

## 4. Discussion

Substitution urethroplasty techniques have progressed significantly, with notable improvements in tissue grafting methods. The ventrolateral aspect of the tongue offers mucosal segments up to 7 – 8 cm in length, depending on the individual’s tongue size, and is readily accessible. Both buccal and lingual mucosas share an embryologic origin, making them easy to harvest with desirable immunologic properties, such as infection resistance. In addition, their structural characteristics – thick epithelium, abundant elastic fibers, thin lamina propria, and dense vascular networks – support successful graft integration through effective imbibition, inosculation, and revascularization processes.^5^

Consequently, this study was conducted to compare the use of BMG and LMG in managing anterior urethral stricture with an evaluation of surgical outcomes and donor site morbidity.

The study was conducted as a case–control study at Ain Shams University Hospital, focusing on patients with lower urinary tract symptoms due to anterior urethral stricture. The patients underwent urethroplasty using the dorsal onlay technique. Specific exclusion criteria were established, such as a history of oral surgery, oral mucosal diseases, and restricted mouth opening. The study results comprehensively compared BMGs and LMGs for the surgical management of anterior urethral stricture.

In our study, there was no significant difference between BMG and LMG regarding the overall urethral success rates, with failure rates of 13.3% in the BMG group and 6.7% in the LMG group, with both grafts showing comparable efficiencies. The variables analyzed, such as stricture length, graft dimensions, and stricture location, showed no significant differences between the BMG and LMG groups. The average stricture length was shorter in the BMG group (7.6 cm) compared to the LMG group (8.3 cm), but this difference was not statistically significant. Similarly, graft length and width were nearly identical across both groups, with no notable differences in the surgical techniques employed, such as the choice of one-step versus two-step urethroplasty. These results suggest that, technically, either graft can be used with similar success rates in repairing the urethral stricture.

Across all studies, both BMG and LMG yielded similar success rates in urethroplasty, with no significant statistical differences. In Lumen *et al.*,^6^ the success rate for LMG was 89.7%, while BMG achieved 82.8%, showing only a minor difference. Al Mamun *et al*.^1^ reported success rates of 80% for LMG and 75% for BMG, again without significant differences. In Pal *et al.*,^7^ the rates were similarly high, with 86% for BMG and 83% for LMG. These findings align with Chauhan *et al.*,^8^ who found a slightly higher success rate for LMG at 80%, compared to 69.2% for BMG, although this difference was not statistically significant.

In addition, our study observed a mean post-operative Q_max_ of 16.4 ± 6.4 mL/s in the BMG group and 18.3 ± 6.5 mL/s in the LMG group. This slight improvement in the LMG group was not statistically significant, which aligns with Pal *et al*.^7^ and Kumar *et al*.^9^ Both studies showed significant improvements in Q_max_ for both groups postoperatively but did not report significant differences. For instance, Pal *et al*.^7^ reported Q_max_ improvements from 8.6 mL/min to 29.56 mL/min for BMG and 7.43 mL/min to 30.29 mL/min for LMG. These findings indicate that both grafts significantly improve urinary flow, with no clear advantage for either.

Regarding donor site morbidity, our study highlighted fewer complications at the donor site in the LMG group. LMG patients experienced fewer issues with drinking, eating soft and solid food, and overall oral functionality. For example, problems with drinking were reported in 10% of LMG patients and 36.7% of BMG patients by day 3. These results are in agreement with other studies. Lumen *et al*.^6^ reported similar outcomes, noting fewer long-term donor-site complications in LMG, though early complications like speech impairment and dysgeusia were more frequent with LMG. Kumar *et al*.^9^ and Chauhan *et al*.^8^ also found that LMG patients had fewer long-term complications, such as scarring, tightness of the mouth, and salivatory disturbances. Overall, LMG consistently demonstrates fewer long-term complications, making it a preferable option in terms of donor-site morbidity.

Regarding speech and oral functionality, our study found that speech and oral functionality were more impaired in LMG patients in the early post-operative phase, particularly on day 3 and week 2. At day 3, 86.7% of LMG patients reported speech problems, compared to only 33.3% of BMG patients. These numbers had improved by week 2, but 53.3% of LMG patients still experienced issues, compared to 20% in the BMG group. This aligns with the findings of Lumen *et al.*,^6^ who reported that 93.1% of LMG patients had speech impairment at day 3, compared to 55.2% of BMG patients. Dysgeusia (taste disturbance) was also more common in LMG patients in our study, with 63.3% affected at day 3, compared to only 13.3% of BMG patients. These results mirrored Lumen *et al*.’s^6^ study, where dysgeusia affected 48.3% of LMG patients versus 13.8% of BMG patients. However, by 6 months, most patients in both groups had recovered, and long-term differences were minimal. While LMG is associated with more early post-operative speech and taste disturbances, these complications generally resolve over time.

Pain and sensitivity disorders at the donor site are important factors in evaluating the recovery process. In our study, oral tightness and sensitivity disorders were significantly more common in the BMG group. At month 6, 26.7% of BMG patients reported oral tightness, compared to only 6.7% of LMG patients. This aligns with findings from Lumen *et al.*,^6^ who reported a higher prevalence of long-term sensitivity disorders in BMG patients (44.8%) compared to LMG patients (31%). Kumar *et al*.^9^ also observed higher rates of long-term complications like perioral numbness and persistent pain in BMG patients. These results suggest that, while both grafts cause some post-operative discomfort, LMG is associated with a quicker recovery and fewer long-term complications related to sensitivity and oral tightness.

In terms of stricture recurrence, our study found a recurrence rate of 13.3% for BMG and 6.7% for LMG, with no significant difference between the two groups. These results are comparable to those from Kumar *et al*.^9^ and Pal *et al*.^7^, both of which reported low recurrence rates (7 – 10%) for both graft types. Similarly, Lumen *et al*.^6^ found no significant difference in recurrence between BMG and LMG, with overall recurrence rates around 10 – 15%. This suggests that both graft types are equally effective in maintaining urethral patency long-term, with low recurrence rates.

Compared with the broader body of research, our study’s findings are largely consistent with those reported in the literature. Both BMG and LMG are effective for managing anterior urethral stricture, with similar success rates and improvements in urinary flow. However, LMG tends to result in fewer long-term donor-site complications, making it a preferable choice for patients concerned about oral morbidity. While LMG may present more short-term complications related to speech and dysgeusia, these issues are typically resolved by 6 months. Overall, LMG offers a slightly better profile regarding donor-site morbidity, while both grafts are equally effective for urethral reconstruction.

The findings of this study have fair clinical implications for surgeons performing urethroplasty for anterior urethral strictures. The choice between BMG and LMG should be based not only on the graft’s technical feasibility but also on the expected donor site morbidity. For patients where early oral function is critical, BMG may be the preferred choice to minimize complications with drinking, eating, and speaking. However, LMG may suit patients who can tolerate short-term oral complications but wish to avoid long-term issues like oral tightness. This tailored approach could improve patient outcomes and satisfaction by aligning surgical decisions with individual patient needs and preferences.

One of the study’s main strengths is its case–control design, which allows a direct comparison between two commonly used mucosal grafts for urethral stricture repair. The study also carefully controlled for confounding factors by ensuring similar baseline characteristics between the two groups, making the results more robust and reliable. In addition, the study provides a comprehensive analysis of both surgical outcomes and donor site morbidity, offering a balanced view of the benefits and drawbacks of each graft type.

## 5. Limitations

Several limitations in this study should be considered when interpreting the findings. One key limitation is the relatively small sample size, with only 30 participants in each group, which may impact the broader applicability of the results. In addition, the follow-up period was limited to 6 months, which may not capture the full range of long-term complications or stricture recurrence rates. Moreover, the study did not include patient-reported outcomes regarding quality of life, which could provide a more holistic view of the impact of donor site morbidity. Finally, excluding certain patient populations, such as those with oral diseases or neurological disorders, may limit the applicability of the findings to broader clinical settings.

## 6. Conclusion

The study concludes that BMG and LMG are effective options for the surgical management of anterior urethral strictures. The surgical outcomes, including graft success, stricture recurrence, and urethral function, were similar between the two groups. However, significant differences were observed in donor site morbidity. Patients in the LMG group experienced earlier post-operative complications related to oral functions, such as difficulties with drinking, eating, and speaking. On the other hand, BMG patients reported more long-term issues with oral tightness. These findings suggest that both grafts are suitable for urethral stricture repair. However, the graft choice may depend on individual patient circumstances, particularly their tolerance for specific oral complications.

## Data Availability

Data will be made available on reasonable request to the corresponding author.
